# Anti‐Icing Organogel Enables Quasi‐Homogeneous Supercooling Preservation of Mouse Hearts

**DOI:** 10.1002/advs.202506968

**Published:** 2025-07-01

**Authors:** Junhao Li, Wei Wang, Chenghao Li, Lintao Kuang, Zhi Huang, Xing Chen, Zhenghao Guo, Kang Liu, Jinping Liu

**Affiliations:** ^1^ MOE Key Laboratory of Hydraulic Machinery Transients School of Power and Mechanical Engineering Wuhan University Wuhan Hubei 430072 China; ^2^ Department of Cardiovascular Surgery Zhongnan Hospital of Wuhan University Wuhan Hubei 430071 China; ^3^ Hubei Provincial Engineering Research Center of Minimally Invasive Cardiovascular Surgery Wuhan Hubei 430071 China; ^4^ Wuhan Clinical Research Center for Minimally Invasive Treatment of Structural Heart Disease Wuhan Hubei 430071 China

**Keywords:** anti‐icing organogel, heart transplantation, ice nucleation, organ preservations, quasi‐homogeneous

## Abstract

Supercooling preservation holds great promise for extending the storage limits of organs. However, supercooled systems are susceptible to stochastic ice nucleation, which can cause fatal damage to the organs. In this study, an organogel interface composed of nanoscale polydimethylsiloxane and dimethyl‐silicone oil is proposed, which presents a significant energy barrier for ice nucleation, comparable to that of homogeneous nucleation. The organogel effectively eliminates primary ice nucleation sites, enabling a quasi‐homogeneous supercooling preservation system that does not rely on cryoprotectant agents or machine perfusion. Through a series of statistical experiments, this approach is demonstrated to be able to maintain stable supercooling and preserve mouse hearts at −4 °C for up to 72 h. A comprehensive assessment conducted at multiple scales indicates that the 36‐h supercooling preservation at −4 °C significantly mitigates cardiac injury by regulating mitochondrial structure and reducing metabolic rates. Utilizing a heart transplantation model with prognostic evaluations extending up to 3 months post‐transplantation, supercooling preservation within the quasi‐homogeneous system is confirmed, which can double the storage duration compared to clinically applied hypothermic preservation methods.

## Graphical abstract

An organogel interface with a large energy barrier for ice nucleation is proposed to enable a quasi‐homogeneous supercooling preservation for organs. This method does not rely on cryoprotectant agents or machine perfusion and can double the ex vivo storage time of mouse hearts.

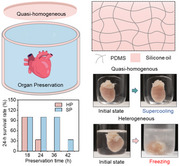

## 1 Introduction

Organ transplantation is regarded as the optimal therapy for the treatment of end‐stage organ failure. However, the advancement of transplantation practices is facing a critical crisis of global donor shortage.^[^ ^1^ ^]^ A fundamental factor contributing to this crisis is the lack of effective long‐term organ preservation technologies.^[^ ^2^ ^]^ Currently, clinically utilized preservation methods, such as static controlled hypothermia and machine perfusion systems, provide viable preservation for only a few hours.^[^ ^3^ ^]^ Extending the storage time beyond 24 h would facilitate improved donor‐recipient matching, optimize transplant tolerance protocols, and ensure adequate preparation for recipients.^[^ ^4^ ^]^ These advancements would enhance global organ sharing efficiency and significantly alleviate the donor shortage crisis.

Supercooling preservation has the potential to significantly extend the organ storage limits, as the metabolic rate halves for every 10 °C reduction in temperature, thereby slowing the rate of organ deterioration. ^[^ ^5^ ^]^However, supercooled water is inherently metastable and is susceptible to spontaneous ice nucleation, which can cause catastrophic damage to organs.^[^ ^6^ ^]^ Traditionally, achieving stable subzero preservation has relied heavily on the use of cryoprotective agents (CPAs) to lower the freezing point.^[^ ^7^ ^]^ Berendsen et al. added cryoprotectants in combination with machine perfusion and preserved rat livers at −6 °C for 72 h.^[^ ^8^ ^]^ Subsequently, they applied the approach to human livers and successfully extended the ex vivo lifespan of the organ to 27 h at ‐4 °C.^[^ ^9^ ^]^ For cardiac preservation, clinicians are implementing ex vivo perfusion systems for donor hearts (the Organ Care System) to extend extracorporeal preservation time. However, supercooling preservation approaches for cardiac grafts remain entirely unexplored in clinical practice.^[^ ^10^ ^]^ Que et al. employed PEG and glucose as cryoprotectants to preserve mouse hearts at −8 °C for 144 h, representing the most recent advancement in the field of heart supercooling preservation.^[^ ^11^ ^]^ Despite these advancements, the use of cryoprotectants is not an ideal choice, due to their cytotoxicity and potential adverse effects on human organs.^[^ ^12^ ^]^ For instance, the acute toxicity of dimethyl sulfoxide may induce serious adverse reactions in individual patients.^[^ ^13^ ^]^ Furthermore, machine perfusion is often necessary in these preservation systems, resulting in increased complexity, cost, and operational challenges. These limitations pose significant barriers to clinical translation, particularly for human organs. Therefore, the ideal preservation strategy would involve static storage in a medium containing as few exogenous compounds as possible.

In this work, we propose an organogel interface, which has a large energy barrier for ice nucleation to eliminate primary ice nucleation sites in the supercooling system. The organogel enables a quasi‐homogeneous supercooling preservation that does not rely on cryoprotectant agents or machine perfusion. As demonstrated through a series of statistical experiments, this approach enables stable supercooling of the preservation system, maintaining the organ at −4 °C for up to 72 h. A comprehensive assessment conducted at multiple scales was undertaken to investigate the effects of preservation temperature on organ injury and metabolic changes during storage. Utilizing a heart transplantation model with prognostic evaluations up to three months post‐transplantation, we verified that supercooling preservation within the quasi‐homogeneous system could significantly promote the storage duration compared with clinically applied hypothermic preservation methods.

## 2 Results and Discussion

**Figure 1:**
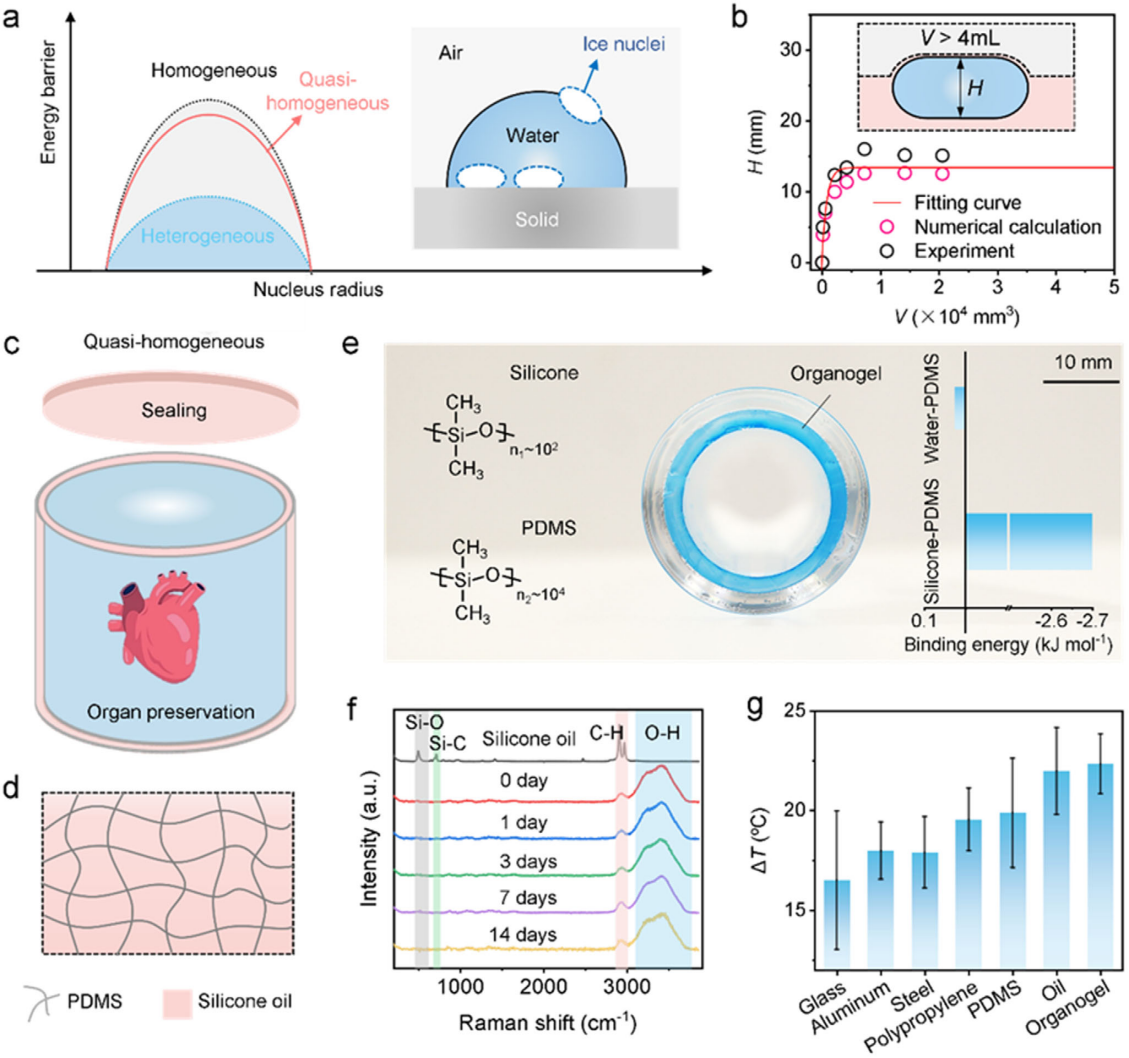
Scheme of quasi‐homogeneous preserver and characterization. a) Energy barriers associated with different types of nucleation. The inset illustrates the schematic diagram of ice nucleation mechanisms of water in a heterogeneous system. The blue circles highlight the primary ice nucleation sites at the liquid‐vapor interface and solid‐liquid interface. b) Equilibrium height H of water suspended at the interface between two different oil layers as a function of water volume *V*. The height *H* reaches a saturated value of ≈13.5 mm when *V* exceeds 5×10^−4^ mm^3^. c). Schematic diagram of the quasi‐homogeneous preserver. d) Microstructure of the organogel interface composed of the PDMS network and confined silicone oil. e) Photographic image of the quasi‐homogeneous preserver. The inset presents physical schemes of the molecular structures of PDMS and oil (left) and binding energies between PDMS and silicone oil or water, calculated using density functional theory (right). f) Evolution of the Raman spectrum of the preservation solution during 2‐week preservation. Raman shift peaks at 500 and 710 cm^−1^ correspond to the Si‐O bond and Si‐C bond in silicone oil, the existence of which indicates the oil contamination in the preservation solution. g) Comparison of the freezing points of water droplets on different surfaces. The oil used in the experiment was phenylmethyl silicone oil.

For conventional supercooling preservation systems, there are two primary mechanisms for ice nucleation: homogeneous and heterogeneous nucleation. Homogeneous nucleation occurs through the random aggregation of interior water molecules, whereas heterogeneous nucleation is catalyzed by substrates or foreign objects. Homogeneous nucleation has a higher energy barrier compared with heterogeneous nucleation; consequently, water freezing is generally initiated by heterogeneous nucleation. Previous studies have identified the water/solid and water/air interfaces as the primary nucleation sites (**Figure** **1**a).^[^ ^14^ ^]^ To suppress heterogeneous nucleation, previous research has tried to suspend small droplets in oil (Figure 1b).^[^ ^15^ ^]^ This approach effectively elevates the energy barrier of nucleation close to the level of homogeneous nucleation. This oil‐encapsulated system can be taken as a quasi‐homogeneous system.

However, this oil‐encapsulated quasi‐homogeneous system is not suitable for organ preservation. Our numerical analyses (Experimental Section) and experimental images demonstrate that the water droplet in oil approaches a flattened shape as the volume of water increases (Figures S1 and S2, Supporting Information). The maximum height is limited to ≈13.5 mm, which is insufficient for large‐sized organ preservation (Figure 1b).^[^ ^16^ ^]^ The flowable and deformable nature of liquid oil allows the water to adapt its shape under the influence of gravity, and comes as the primary challenge for the formation of a large‐scale quasi‐homogeneous environment. To address these problems, we propose a novel design with a semi‐solid organogel. The organogel is composed of Polydimethylsiloxane (PDMS)‐network and dimethyl‐silicone oil (Figure 1c,d).^[^ ^17^ ^]^ The organogel creates an icephobic interface that can be architected into arbitrary sizes and shapes. Also, the organogel can be affixed to solid walls to form rigid containers. These features distinguish the PDMS‐oil composite surface from conventional rigid and flat anti‐icing organogels reported in prior studies ^[^ ^18^ ^]^ while also addressing limitations in organ preservation applications—an area never explored in earlier research. The adaptability and structural versatility of this surface highlight its unique suitability for practical deployment. Details of the fabrication process are provided in the Experimental Section and Figure S3, Supporting Information. The PDMS chains exhibit strong hydrophobic interactions with the oil, as indicated by the binding energy shown in Figure 1e. Additionally, the Raman spectrum in Figure 1f confirms that the PDMS network securely binds the oil, preventing it from leaking into the preservation liquid (University of Wisconsin solution, UW solution) over a period of two weeks. Moreover, the organogel demonstrates good mechanical stability when immersed in the UW solution (Figure S4, Supporting Information). Most importantly, the organogel exhibited anti‐freezing properties comparable to those of liquid oil. The surface freezing point of the water droplet is as low as −22.5 °C (Figure 1g). The freezing delay times of droplets on organogel surfaces are significantly longer than those on glass surfaces (Figure S5, Supporting Information). Collectively, these characteristics guarantee the reliable performance of the container as a quasi‐homogeneous system.

**Figure 2:**
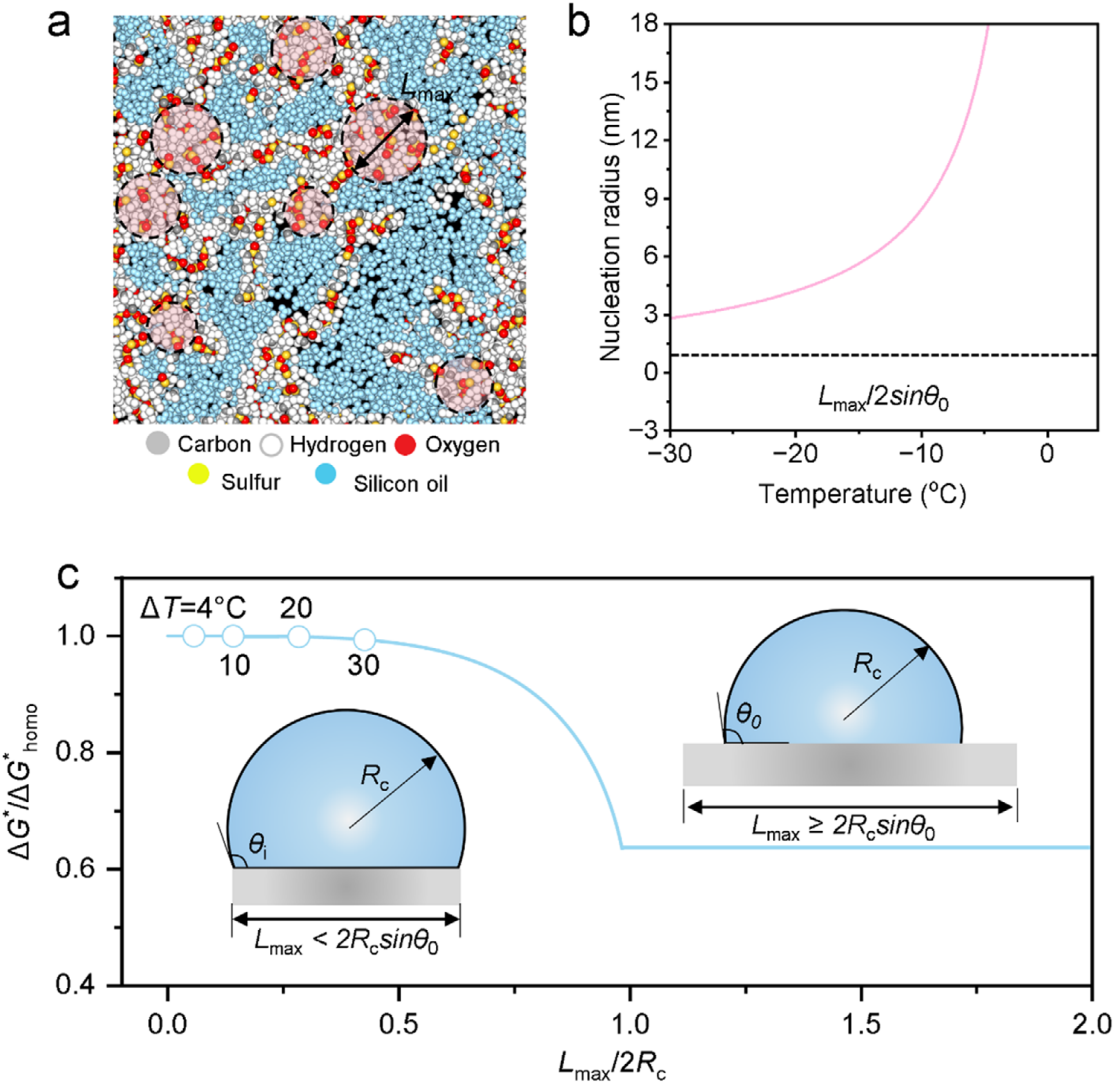
Micro‐scale mechanism of ice nucleation on the organogel surface. a) Distribution of PDMS and silicon oil on the organogel interface. The circle highlights the maximum diameter of the ice nucleation area. b) Ice nucleation radius as a function of the temperature. c) Free‐energy barrier of ice nucleation on the organogel surface versus the normalized size of the organogel microstructure. The inset figure shows the physical model of the ice nucleus on a nano‐confined surface.

The icephobic property of a surface fundamentally arises from the complex interactions between ice and the surface, which are governed by two critical factors: surface energy and microstructure. As illustrated earlier, the organogel surface comprises a PDMS network infused with silicone oil. The oil phase exhibits low surface energy and imposes a substantial energy barrier for ice formation that is near the homogeneous nucleation regime. In contrast, PDMS displays a lower energy barrier and nucleates ice at higher temperatures (Figure 1g). However, the organogel surface demonstrates anti‐freezing performance akin to pure oil. To elucidate the mechanism, we conducted molecular dynamics simulations to explore the microstructure of the organogel interface. As depicted in **Figure** **2**a, the PDMS network displays a loose configuration, interspersed with silicone oil. Owing to its lower nucleation energy barrier, ice preferentially nucleates within the PDMS regions, where it becomes confined by the polymer boundaries. The maximum diameter of the circular area on PDMS, denoted as *L*_max_, is ≈2.1 nm, which corresponds to a maximum nucleation radius *R*_max_ of ≈1.1 nm (*R*_max_ = *L*_max_/2sin *θ*_0_, where *θ*_0_ ≈100 ° is the ice contact angle on PDMS). This radius is smaller than the critical nucleation radii *R*_c,_ across the temperature range from −30 to 0 °C (Figure 2B), which makes the organogel surface difficult for ice nucleation.

Using classical nucleation theory,^[^ ^15^, ^19^ ^]^ we quantitatively analyzed the energy barrier for ice nucleation on the organogel surface. Based on the physical model of ice nucleation inside Figure 2c, the relationship between the ratio of the energy barrier for heterogeneous nucleation to that of the homogeneous nucleation, ΔG*/ΔG*_homo_ and *L*_max_/2*R*_c_ can be expressed as
(1)

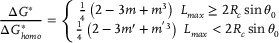

where *m* ≈ cos*θ*_0_, *m*“ ≈ cos*θ*_i_. A transition occurs at *L*_max_/2*R*_c_ = sin*θ*_0_, accompanied by a zero‐sized effect^[^ ^20^ ^]^. As illustrated in Figure 2c, for *L*_max_ ≥ 2*R*_c_ sin*θ*_0_, the contact angle of the ice nucleus is governed by Young's equation γ_WI_cos*θ*_0_ = *γ*_WO_ – *γ*_IO_, where *γ*_WI_, *γ*_WO_, *γ*_IO_ are the interfacial surface tensions of water‐ice, water‐organogel, and ice‐organogel, respectively. Conversely, *L*_max_ < 2*R*_c_ sin*θ*_0_, the contact angle increases to *θ*_i_ = arcsin (*L*_max_/2*R*_c_) as the surface size constraint distorts the shape of the ice nucleus. *θ*_i_ is obviously larger than *θ*_0_, elevating the free energy barrier for ice nucleation. As *L*_max_/2*R*_c_ approaches zero, the free energy barrier asymptotically approaches that of homogeneous nucleation. Noticeably, the organogel surface exhibits a high nucleation barrier close to that of homogeneous nucleation at the supercooling from 0 to 30 K, which renders it unlikely to serve as a nucleation site for ice crystals.

Ice nucleation is a stochastic process, and it is currently convenient to describe this process as a non‐homogeneous Poisson process, as the nucleation rate may not remain constant over time. The non‐homogeneous Poisson distribution has been used to characterize nucleation in metals,^[^ ^21^ ^]^ phase‐change materials^[^ ^22^ ^]^ and in the solutioncrystallization process.^[^ ^23^ ^]^ To predict the performance of organ preservation in such a quasi‐homogeneous system, we first measured the supercooling degree of a small‐volume droplet and subsequently developed a statistics‐based theoretical model to quantitatively predict the supercooling performance of a large‐scale preserver.^[^ ^22^ ^]^ The supercooling degree was measured with a statistical method (Experimental Section). We conducted 100 cycles of measurements to generate a freezing survival function (Figures S10 and S11, Supporting Information), which is defined as the probability of samples that remain unfrozen at a supercooling degree of Δ*T*. We can express the survival function with an independent variable of Δ*T* as

(2) $$\chi \left(\Delta T\right)=e^{-\frac{V}{\beta }\int_{0}^{\Delta T}J\left(\Delta T\right)d\Delta T}$$

from the survivor function, the nucleation rate *J (T)* as a function of temperature *T* was fitted to the power law, *J (T)* can be expressed as

(3) $$J\left(T\right)=\gamma \Delta T^{n}$$

where *γ* and *n* are two empirical parameters related to the surface properties of the organogel in this study. Since the preserver interface serves as the primary nucleation site, the degree of supercooling can be expressed as a function of the surface area of the preserver, *S*

(4) $$\Delta T=k^{\frac{1}{n+1}}{\left(\frac{n+1}{\gamma S}\right)}^{\frac{1}{n+1}}\Gamma \left(\frac{n+2}{n+1}\right)$$

where *k* is the cooling rate and Γ is the gamma function. This equation establishes a correlation between experimentally observed supercooling in small samples and expected performance in larger systems. Figure S10b (Supporting Information) shows a good agreement between the predicted results and experimental data. The preservation time for a given supercooling degree can also be predicted as

(5) $$t=\frac{1}{SJ\left(T\right)}$$

**Figure 3:**
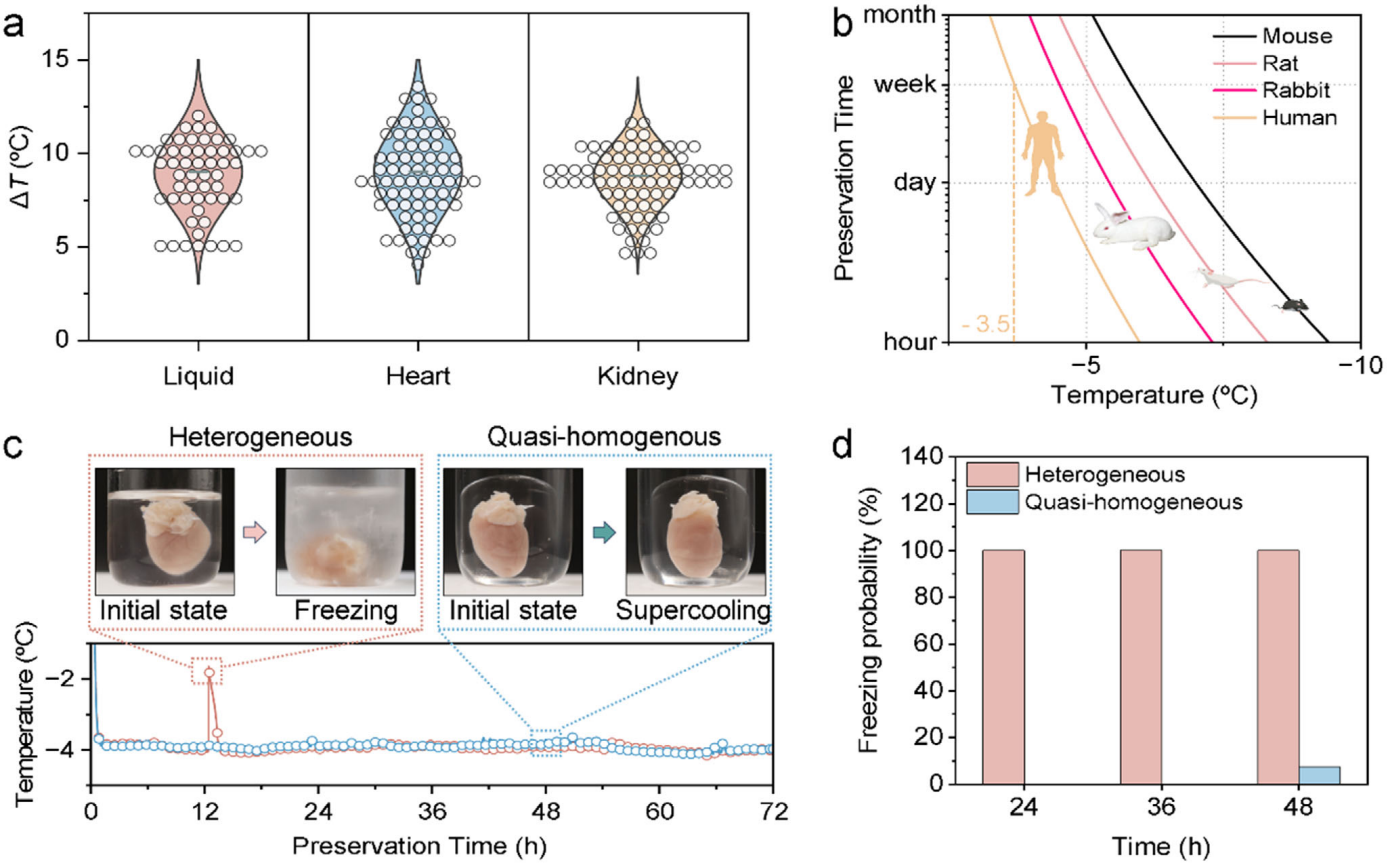
Supercooling performance of quasi‐homogeneous preserver. a) Impact of adding rat organs on the supercooling degree of the preservation solution, demonstrating how the presence of biological material affects supercooling performance. b) Statistical prediction of unfrozen liquid temperatures for various preservation durations of typical organs from animals and humans. The organs are assumed to have the same surface area as the preservation container, which is modeled as spherical. c) Variations in preservation temperatures during long‐term preservation of a mouse heart. The inset figures illustrate the changes in the liquid state at specific times. In the heterogeneous system, a significant temperature increase occurs ≈12 h, indicating that freezing has occurred. In contrast, the preservation solution remains in a liquid state for 72 h in the quasi‐homogeneous system. d) Freezing probabilities of supercooling preservations at −4 °C over different preservation time.

In a system with organs, the organ/water interface could possibly enhance heterogeneous nucleation. To investigate the effect of organs on the supercooling of our system, we put rat hearts and kidneys in the preservation system and compared the results with those of the pure liquid system. As shown in **Figure** **3**a, the inclusion of rat hearts and kidneys has little impact on the supercooling degree. The results prove that the energy barrier of nucleation at the organ/water interface is close to or larger than that at the organogel/water interface. Raman spectroscopy of the organ surface (Figure S12, Supporting Information) further reveal abundant lipid content, which may function as a cryoprotectant by inhibiting ice crystal formation.^[^ ^24^ ^]^ Hence, we can evaluate the preservation time using the supercooling model above.

Figure 3b illustrates the preservation time at different temperatures for typical animal or human organs. In such a quasi‐homogeneous system with organs, safe preservation of human organs can be maintained at temperatures below −3 °C for up to one week. For the rat heart, the theoretical preservation limit can be up to −5 °C. To verify the prediction, we stored a rat heart in an organogel‐encapsulated container and a glass container at −4 °C, respectively. As shown in Figure 3c, the quasi‐homogeneous system maintains a supercooled state for more than 72 h. While in the heterogeneous system, freezing occurs after just 12 h of preservation. Through 50 rigorously independent replicate experiments that showed excellent reproducibility, we observed no freezing in the quasi‐homogeneous system during a 36‐h preservation period. Even in the period of 48 h, the freezing rate is only 7.5%. In stark contrast, all samples in the heterogeneous system experience freezing within 24 h (Figure 3d). These findings confirm that the quasi‐homogeneous system offers the possibility for long‐term supercooled organ preservation.

**Figure 4:**
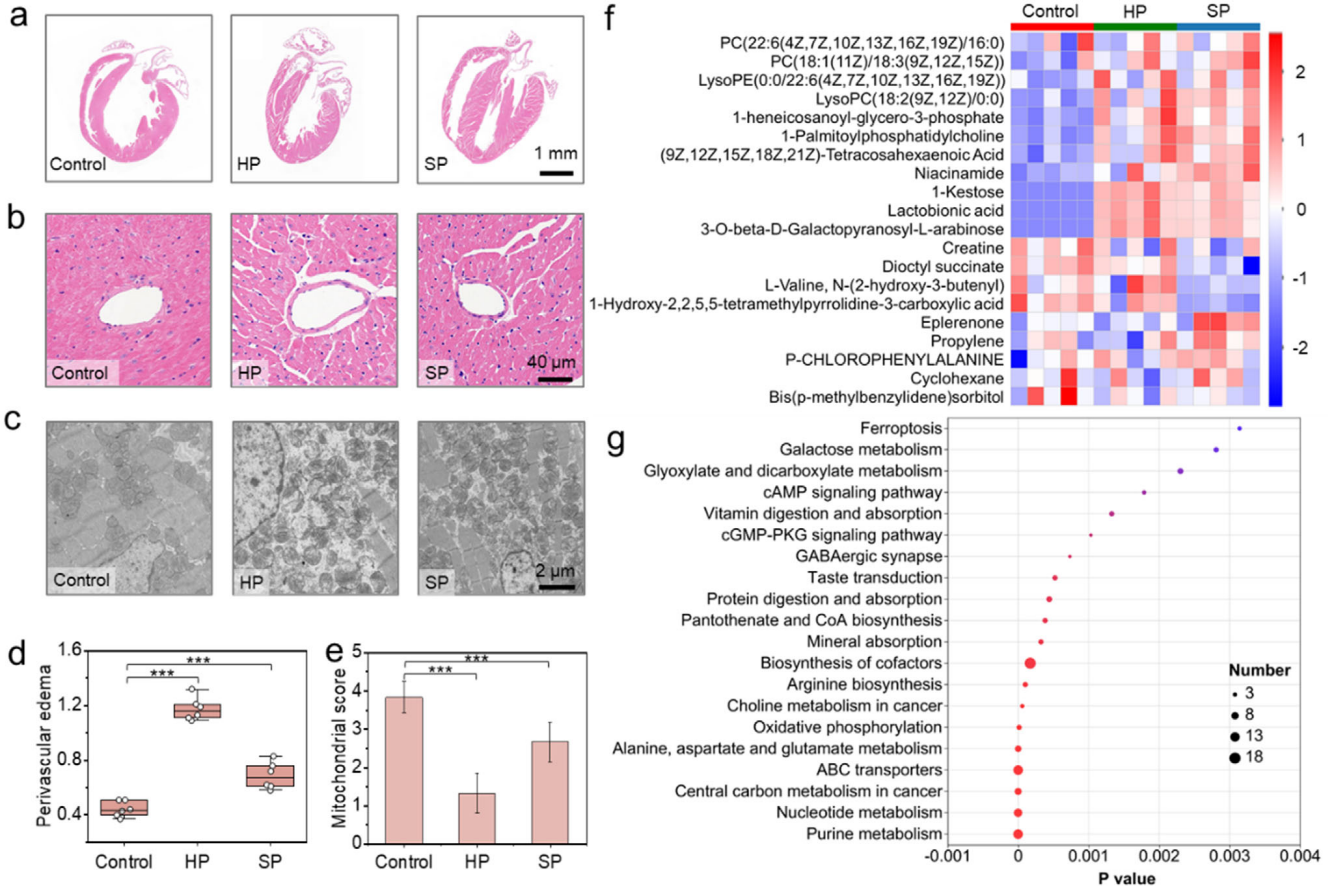
Multi‐level assessment of myocardial preservation injury after 24‐h preservation. a) Representative microscopy images of whole hearts and b) myocardial tissue from the control, HP (hypothermic preservation), and SP (supercooling preservation) groups stained with hematoxylin and eosin. c) Changes in microstructure (mitochondria) of cardiomyocytes assessed by transmission electron microscopy. d) Degrees of perivascular edema in the control, HP, and SP groups. The edema degree was calculated by subtracting the vascular area from the total area of perivascular edema and then dividing the result by the vascular area. e) Mitochondrial scores across the three groups. The scores were graded on a scale of 1 to 4 based on mitochondrial damage, following the criteria established by Gabriel Amir. f) Top 20 molecules with significant changes detected by untargeted metabolomics after 24 h of preservation, highlighting key metabolites affected by preservation methods. g) Top 20 KEGG (Kyoto Encyclopedia of Genes and Genomes) signaling pathways enriched by the significantly changed molecules. Data are mean ± SD, with *n* = 6 for each group. Statistical comparisons between groups were performed using ANOVA tests, with significance indicated by ***p < 0.01.

We conducted a 24‐h preservation study on mouse hearts using the quasi‐homogeneous preserver and evaluated myocardial injury across multiple scales, including gross structure, macroscopic pathology, microstructure, and molecular expression. Notably, machine perfusion is not necessary in the absence of cryoprotective agents (CPAs), which renders the preservation procedure more streamlined compared with previous reports.^[^ ^8^ ^]^ As shown in **Figure** **4**a, hearts by hypothermic preservation (HP) at 4 °C present obvious thickened myocardium and a smaller cavity of left ventricular cavity, while supercooling preservation (SP) at −4 °C maintains similar heart structures as compared with the control group (no preservation). Hematoxylin‐eosin (HE) staining was employed to assess myocardial edema, a critical diagnostic marker of tissue ischemic damage. Results presented in Figure 4a,b indicate that hearts by HP exhibit significant edema of cardiomyocyte and perivascular (1.175 ± 0.085) with indistinct intercellular spaces. In contrast, hearts preserved via SP demonstrate remarkable improvement in cardiomyocyte and perivascular edema (0.687 ± 0.099, *p* < 0.05) (Figure 4b,d). At the microstructural level, the mitochondrial matrix in the HP group is severely compromised, exhibiting broken ridges and substantial membrane destruction in some mitochondria, with most mitochondrial scores falling within grades 1–2 (1.34 ± 0.52). The SP group displays some degree of edema and increased ridge spacing; however, the membranes remain intact, and the edges of the mitochondria are clear. Mitochondrial scores are primarily in grades 2–3 (2.67 ± 0.52, *p *< 0.05) (Figure 4c,e). At the molecular level, metabolomics analysis reveals a total of 255 metabolites that exhibit significant changes following preservation. Notably, over one‐third of these differential metabolites (86 out of 255) are enriched in various metabolic functions, including amino acids, monosaccharides, phospholipids, carboxylic acids, etc. (Figure S13, Supporting Information). The top 20 metabolites were predominantly associated with metabolic pathways, including oxidative phosphorylation, purine metabolism, nucleotide metabolism, as well as alanine, aspartate, and glutamate metabolism (Figure 4f,g). Among these pathways, oxidative phosphorylation, which serves as the primary driver of ATP synthesis, underpins both mitochondrial function and quality control mechanisms.^[^ ^25^ ^]^ Concurrently, other metabolic pathways play critical roles in safeguarding mitochondrial integrity.^[^ ^26^ ^]^ Collectively, these findings underscore that supercooling preservation significantly attenuates cardiac preservation injury by modulating mitochondrial structure and preserving metabolic homeostasis. These further highlight that targeting metabolic regulation emerges as a promising avenue for future research, with the potential to further mitigate mitochondrial and myocardial damage and thereby enhance the efficacy of supercooling preservation.

**Figure 5:**
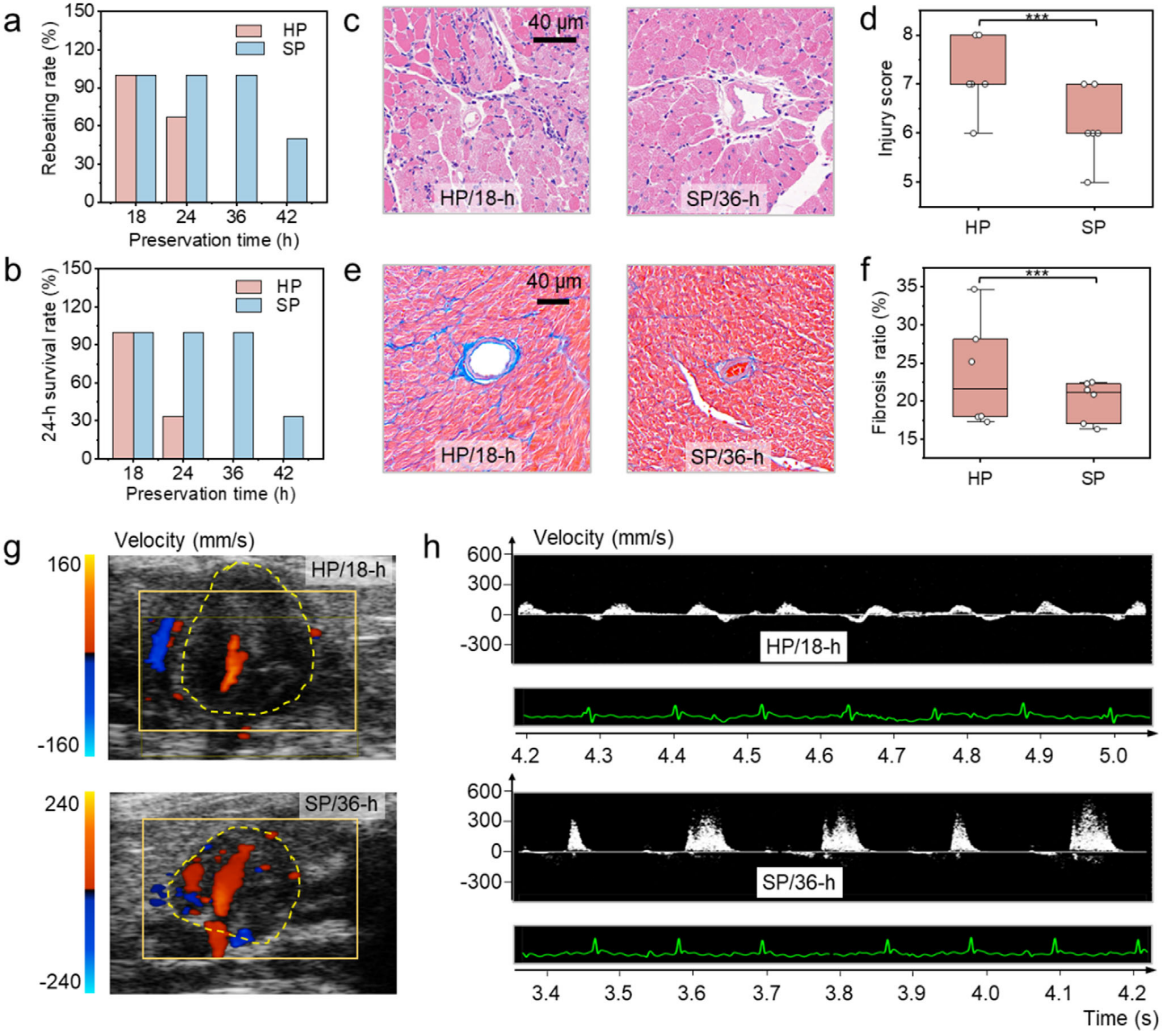
Evaluation of short‐term and long‐term prognosis after heart transplantation. a) Rebeating rates after heart transplantation. b) Survival rates 24 h post‐transplantation for various preservation times (*n *= 6). c) Microscopy images of hearts stained with hematoxylin and eosin 24 h after transplantation. d) Pathological injury scores after transplantation, calculated based on the degree of cardiomyocyte necrosis, hemorrhage, edema, and neutrophil infiltration (*n* = 6). e) Microscopy images of myocardial fibrosis observed 3 months post‐transplantation, stained by Masson. f) Fibrosis ratios after 3 months post‐transplantation, measured using the Fiji software program according to established protocols (*n *= 6). g) Echocardiography images of cardiac grafts 3 months post‐transplantation (*n* = 3). The red region denotes blood inflow, while the blue region indicates blood outflow. h) Velocity of blood flow in the heart graft (*n* = 3). Data are mean ± SD. Statistical comparisons between groups were performed using ANOVA tests.

To further evaluate organ function following transplantation, we established a heterotopic heart transplantation model (Experimental Section; Figure S14, Supporting Information), which serves as the gold standard for assessing the effectiveness of organ preservation. Compared to the HP group, the rebeating rates (**Figure** **5**a) and 24 h survival rates (Figure 5b) in the SP group are significantly improved. Hearts from mice could be preserved for up to 36 h with a 100% 24‐h survival rate using SP. This maximum storage duration (with a 100% 24‐h survival rate) is two times the value achieved by the HP method (18 h). We subsequently compared organ injury post‐transplantation between the group by HP for 18 h and SP for 36 h. The heart rates are both ≈300–350 beats per minute, while the rebeats of hearts by SP have a greater contraction, as can be seen in Movie S1and S2. Pathological damage was assessed and scored based on the extent of cardiomyocyte necrosis, hemorrhage, edema, and neutrophil infiltration. Although both groups exhibit significant tissue injury 24 h after transplantation, the injury score in the SP group is slightly lower (7.17 ± 0.75 vs 6.0 ± 0.89, *p* > 0.05) (Figure 5c). Additionally, we also compared the pathological damage observed 24 h after transplantation with HP and SP methods at different preservation times. The pathological damage with the supercooling method is always lower than that with hypothermic preservation (Figure S15, Supporting Information), which further highlights the importance of supercooling.

Most importantly, we observed the long‐term prognosis following transplantation in the context of supercooling preservation. Echocardiography results demonstrate that all mouse hearts survive after 3 months’ post‐transplantation, evidenced by clearly visible heart pulsation and blood flow (Figure 5g; Movie S3 and S4). The blood flow velocity in the SP group is significantly larger than that observed in the HP group (Figure 5h), suggesting a more favorable heart survival status in the SP group. The degree of fibrosis and pathological damage of the HP group is also slightly serious than that of the SP group (23.55% ± 7.04% vs 20.09% ± 2.70%, *p* > 0.05, Figure 5e,f; Figure S16, Supporting Information). Collectively, these results underscore the advantages of longer‐term preservation achieved through the quasi‐homogeneous system compared with traditional hypothermic preservation.

## 3 Conclusion and Perspective

In summary, we propose an organogel interface that can effectively eliminate primary ice nucleation sites to achieve a quasi‐homogeneous supercooling preservation. The system of the preserver and organ achieves a stable supercooling state at −4 °C up to 36 h, which is six times longer than the safe preservation time of the clinical static cold storage. The quasi‐homogeneous system eliminates the need for cryoprotective agents and avoids the complexities and costs associated with machine perfusion, which is quite different from previous supercooling preservation systems. Using the preserver, we demonstrate that supercooling preservation could significantly reduce cardiac injury by regulating mitochondrial structure and depressing the metabolic rate. Further heart transplantation and long‐term prognosis after transplantation confirm that the quasi‐homogeneous system can extend the heart preservation time up to two times as compared with hypothermic preservation. The next step involves achieving supercooling preservation for large animals or even human organs. Given the significantly larger dimensions of such systems compared to our current murine heart model, resolving stochastic nucleation dynamics and enhancing long‐term stability will be essential. For future applications, integrating advanced characterization techniques (e.g., in situ imaging, cryo‐EM) will further clarify anti‐icing mechanisms at multiple length scales. Additionally, combining bio‐compatible cryoprotectants with optimized ice‐inhibitory performance and minimized toxicity will be key to achieving highly efficient and clinically translatable preservation. Ultimately, these advancements could revolutionize organ preservation practices, enabling broader access to high‐quality organs for transplantation.

## 4 Experimental Section

### Characterizations

The leakage of oil in the UW solution was examined by a laser confocal Raman spectrometer (Alpha 300 RA, Oxford). The elastic modulus of the organogel was measured using an electronic universal testing machine (UTM 2503, Shen Zhen Technology Stock Co. Ltd.). The macrostructure of the cardiac tissue was scanned and observed (Pannoramic MIDI, 3DHISTECH) after hematoxylin‐eosin (HE) staining. The mitochondrial structure of cardiac cardiomyocytes was examined by transmission electron microscopy (Hitachi H‐7650, Hitachi). The echocardiography was performed 3 months after heart transplantation using an ultrasound scanner (VINNOD860LAB, VINNO) by a doctor who was blind to the experimental design. The myocardial fibrosis after 3 months of transplantation was scanned and observed (Pannoramic MIDI, 3DHISTECH) after Masson staining and Sirius red staining. The fibrosis degree was measured using the Fiji software program.

### Numerical Simulation of Water Geometry Suspended in Oil

COMSOL Multiphysics software was employed to simulate the two‐dimensional equilibrium state of water suspended at the interface between two oil layers (Figure S1, Supporting Information). The densities of the light oil (A), water (B), and dense oil (C) were set to 930, 1000, and 1100 kg m^−^^3^, respectively. The dynamic viscosities were 0.01, 0.15, and 0.00101 Pa s for oils A, B, and C, respectively. The interfacial surface tensions for the pairs A‐B, B‐C, and A‐C were set at 0.02, 0.02, and 0.025 N m^−1^, respectively. The contact angles at the three‐phase interface were defined as 90°. The simulation utilized the “Laminar Flow” module and the “Ternary Phase Field” module. Initially, circular droplets with diameters of 3, 5, 8, 12, 15, and 17 mm were positioned between the two oil layers. The droplets spread under the combined effects of interfacial surface tension and gravity, ultimately reaching an equilibrium shape.

### Fabrication of Quasi‐Homogeneous Preserver

The quasi‐homogeneous preserver consisted of an open organogel container and a sealing lid. The open container was fabricated by coating an organogel layer on the inner wall of a quartz glass vessel (Figure S3, Supporting Information). Initially, the glassware was cleaned with absolute ethanol (> 99.7% purity, Sigma‐Aldrich) and ultrapure water (18.3 MΩ), followed by plasma treatment at a power of 18 W for 10 min. Meanwhile, polydimethylsiloxane (Sylgard 184, Dow Corning) was prepared by mixing the elastomer base and curing agent in a 10:1 ratio. The precursors were thoroughly mixed in a centrifugal mixer at 1000 rpm for 10 min and then degassed for at least 30 min in a vacuum chamber. The mixture was subsequently poured into the glassware and cured at 80 °C. Prior to curing, a drum‐shaped PTFE mold was placed in the center of the glassware. After 2 h of curing, the samples were removed from the mold and infused by submersion in dimethyl silicone oil (viscosity ≈10 mPa s, Sigma‐Aldrich) for at least 24 h. The sealing lid was formed from a free organogel layer, which was removed from a concave cylindrical mold.

### Binding Energies Calculation

Binding energies between PDMS and oil/water (Figure S6, Supporting Information) were determined using Density Functional Theory (DFT) calculations. Prior to the calculations, a global configuration search was conducted to achieve energy convergence of the system using the Forcite module in Materials Studio, employing the COMPASS III force field. The ensemble was configured to NVT, maintaining a constant temperature of 300 K. The timestep was set to 1 fs, with a total simulation duration of 1 ns. The binding energy calculations were performed using Gaussian 16, with the B3LYP functional and the 6–31G(d) basis set employed for structural optimization and frequency calculations. The binding energies of PDMS‐oil (*E*_PDMS‐oil_) and PDMS‐water (*E*_PDMS‐water_) were calculated using the equations:

(6) $$E_{\mathrm{PDMS}-\mathrm{oil}}=E_{\mathrm{tot}-}\left(E_{\mathrm{PDMS}}+E_{\mathrm{oil}}\right)$$

(7) $$E_{\mathrm{PDMS}-\mathrm{water}}=E_{\mathrm{tot}-}\left(E_{\mathrm{PDMS}}+E_{\mathrm{water}}\right)$$

where *E*_tot_ the total energy of the system, *E*_PDMS_, *E*_oil_ and *E*_water_ denote the energies of PDMS, oil, and water, respectively.

### Measurement of Water Freezing Points on Surfaces

The freezing points of water droplets on various surfaces were measured in a closed environment filled with nitrogen gas (Figure S7, Supporting Information). Approximately 10 5‐µL droplets of ultrapure water were placed on different surfaces using transfer pipettes. To prevent evaporation loss, each droplet was covered with a thin layer of silicone oil. The surfaces were adhered to a Peltier cooling plate using thermal silicone grease and were cooled at a rate of 6 °C min⁻¹. The formation of ice was monitored using an optical microscope (M.ZUIKO DIGITAL ED, Olympus) equipped with a digital camera (DMC‐GH4, Panasonic). The temperature at which a sudden change in opacity of the water droplets was first observed was recorded as the nucleation temperature.

### Molecular Dynamics Simulation

The polymerization degree of PDMS and silicone was set as 100 and 8 in the simulation, respectively. Using Material Studio, an amorphous unit cell (105.3 × 105.3 × 98 Å) containing 24 PDMS chains and 192 silicone oil molecules was modeled (Figure S7, Supporting Information). After structural optimization, a vacuum layer with a thickness of 25 Å was added on both ends of the *z*‐axis. The GAFF atom types and corresponding bonded parameters were specified using Sobtop, and MMFF94 atomic charges were employed. Molecular dynamics simulations were performed using GROMACS, with all simulations conducted under the NVT ensemble. Periodic boundary conditions were applied in both *x* and *y* directions, and the cutoff distances for Coulombic and van der Waals interactions were set to 1 nm. After energy minimization, a 50 ns equilibration was carried out using a V‐rescale thermostat, where the reference temperature linearly increased from 0 K to 293.15 K over the first 0 to 30 ps, and then remained constant. The structure in Figure 2a was extracted from the simulation at 50 ns mark.

### Supercooling Measurements

For small droplets, measurements of supercooling degrees were conducted using quasi‐free water droplets suspended at the interface between dimethyl silicone oil (density: 0.96 g cm⁻^3^) and phenylmethyl silicone oil (density: 1.1 g cm⁻^3^), as illustrated in Figure S9a, Supporting Information. For larger volumes of water, the fabricated quasi‐homogeneous preserver was employed (Figure S9b, Supporting Information). It is noteworthy that the liquid oil and organogel interface exhibited nearly equivalent freezing performance for water (Figure 1g), allowing the freezing behaviors of water in both containers to be described using the same empirical parameters, *γ* and *n*. Both devices were immersed in a refrigerated circulating bath. A linear cooling ramp gradually reduced the system temperature at a rate of 6 °C min⁻¹ until nucleation occurred. For small droplets, nucleation was detected in a manner similar to that observed with water droplets on the organogel surface (Figure S9c, Supporting Information). In the case of larger volumes of water, freezing was monitored using a thermocouple. The onset of nucleation was marked by a sudden temperature jump to 0 °C, attributed to the release of latent heat (Figure S9d, Supporting Information). Following this, the frozen sample was heated back to room temperature to complete the cycle. At least 100 freezing cycles were conducted to perform a statistical analysis, with the probability distribution of the supercooling degree illustrated in Figure S10, Supporting Information. Based on the experimental data, the “survival function” in Figure S11 (Supporting Information) was generated.

### Untargeted Metabolomics Analysis

Hearts were collected after preservation for 24 h, and metabolites from a 50 mg sample were extracted. The mixture was then sonicated, precipitated, and centrifuged, with the supernatant carefully transferred to sample vials for LC‐MS/MS analysis. Following the completion of mass spectrometry detection (UHPLC‐Q Exactive system, Thermo Fisher Scientific), the raw data from LC/MS were preprocessed using Progenesis QI software (Waters Corporation, Milford, USA). Subsequently, variance analysis, Student's t‐test, and fold difference analysis were conducted to summarize the differential metabolites between the two groups. These metabolites were then mapped to their biochemical pathways through metabolic enrichment and pathway analysis, utilizing database searches from KEGG (Figure S13, Supporting Information; http://www.genome.jp/kegg/).

### Heart Transplantation Model

Male C57BL/6 mice aged 8 weeks were selected as experimental subjects. All experiments and procedures were approved by the Institutional Animal Care and Use Committee at Zhongnan Hospital of Wuhan University (No. LBSM2024009). The mice were randomly divided into three groups: a normal group, a 4 °C hypothermia preservation group, and a −4 °C supercooling preservation group (Figure S14, Supporting Information). After administering an intraperitoneal injection of pentobarbital sodium (60 mg kg^−1^) for deep anesthesia, the thoracic and abdominal cavities were opened. The heart was obtained by slowly perfusing UW solution through the aorta, followed by ligation of the inferior vena cava and pulmonary veins. The aorta and pulmonary artery were then transected. Myocardial damage was evaluated after preservation. Subsequently, end‐to‐side anastomoses were performed between the donor aorta and the recipient's abdominal aorta, as well as between the donor pulmonary artery and the recipient's subrenal inferior vena cava, establishing an abdominal ectopic heart transplantation model. Graft survival was recorded for each group, and heart graft samples were ultimately collected for further testing.

## Conflict of Interest

The authors declare no conflict of interest.

## Supporting information



Supporting Information

Supplementary Movie1

Supplementary Movie2

Supplementary Movie3

Supplementary Movie4

## Data Availability

Data sharing is not applicable to this article as no new data were created or analyzed in this study.
