# Supplementary material for: Anti‐Icing Organogel Enables Quasi‐Homogeneous Supercooling Preservation of Mouse Hearts
Source: Adv Sci (Weinh). 2025 Jul 1;12(37):e06968. doi: 10.1002/advs.202506968 (PMC12499471; doi:10.1002/advs.202506968)
Supplement: Supplementary file 1 — Supporting Information [file ADVS-12-e06968-s004.docx]

Supporting Information

**Anti-icing Organogel Enables Quasi-homogeneous Supercooling Organ Preservation of Mouse Hearts**

*Junhao Li, Wei Wang, Chenghao Li, Lintao Kuang, Zhi Huang*, Xing Chen, Zhenghao Guo, Kang Liu*, Jinping Liu**


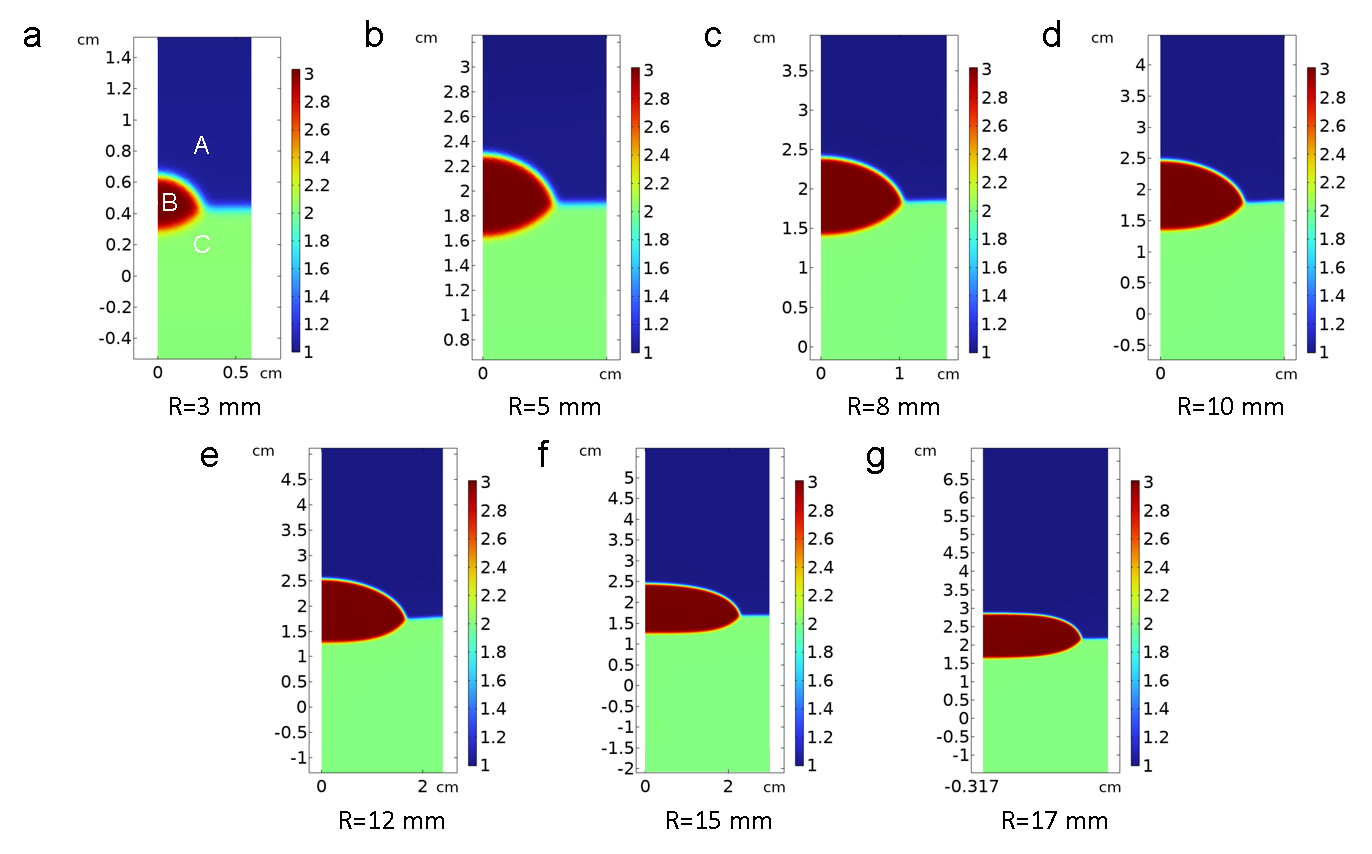


**Figure S1.** Numercial simulation of the equilibrium geometry of water droplets with different radii between two oil layers

**
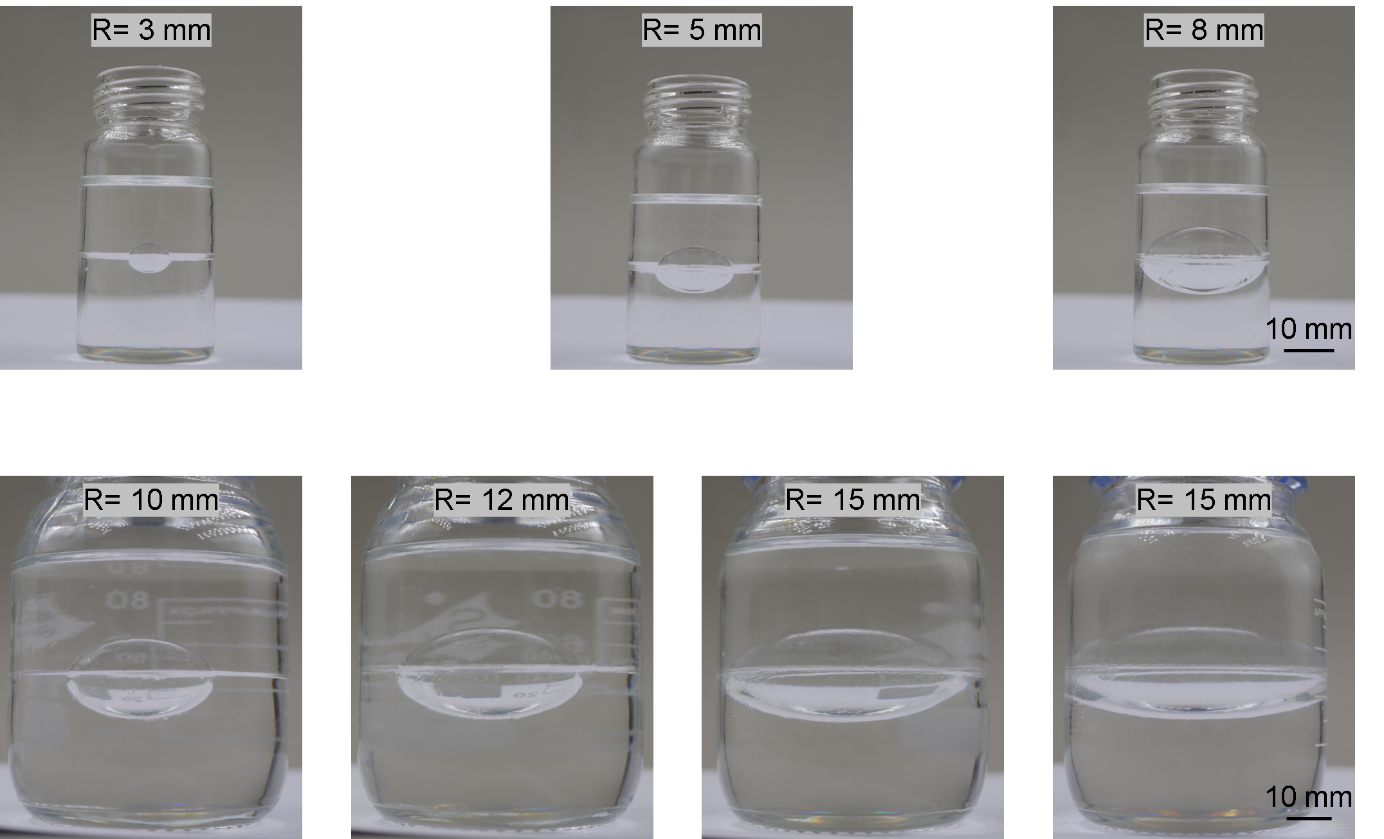

Figure S2.** Experimental images of the equilibrium geometry of water droplets with different radii between two oil layers


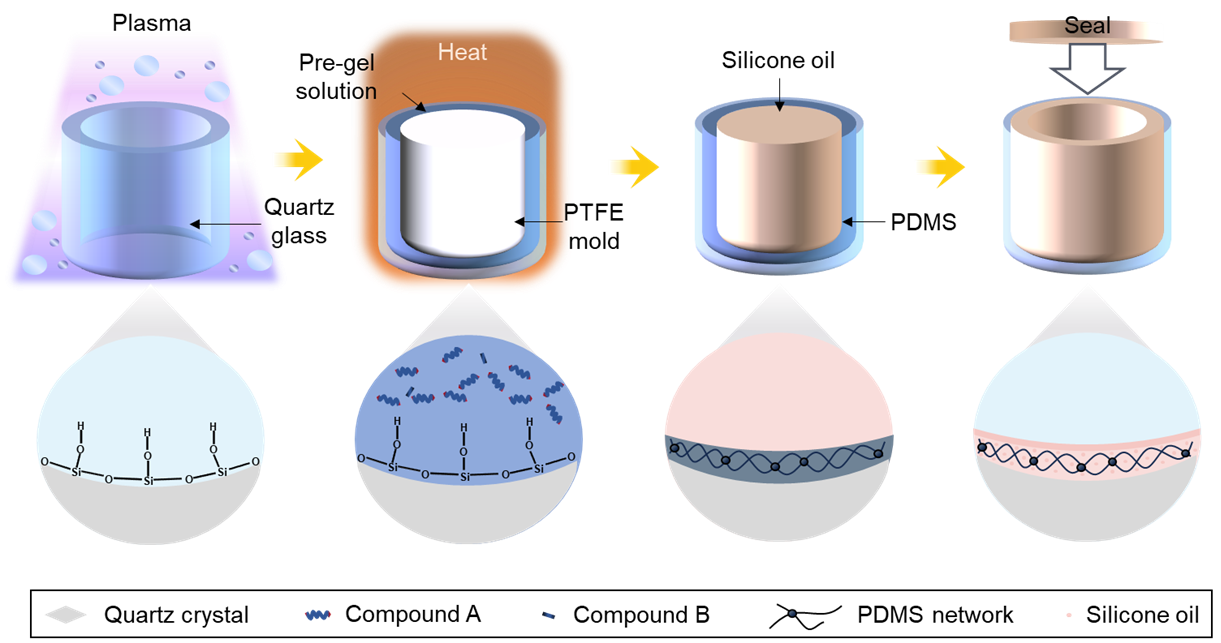


**Figure S3.** Fabrication process of the quasi-homogeneous perserver


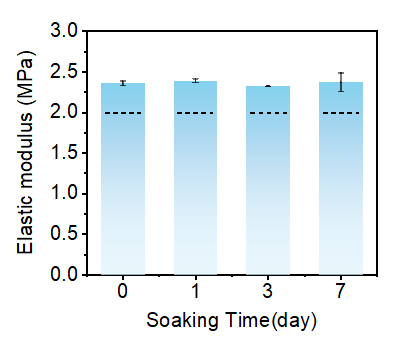


**Figure S4.** Evolution of the elastic modulus of the organogel immersed in the preservation solution. The dotted line represents the elastic modulus of PDMS.

**
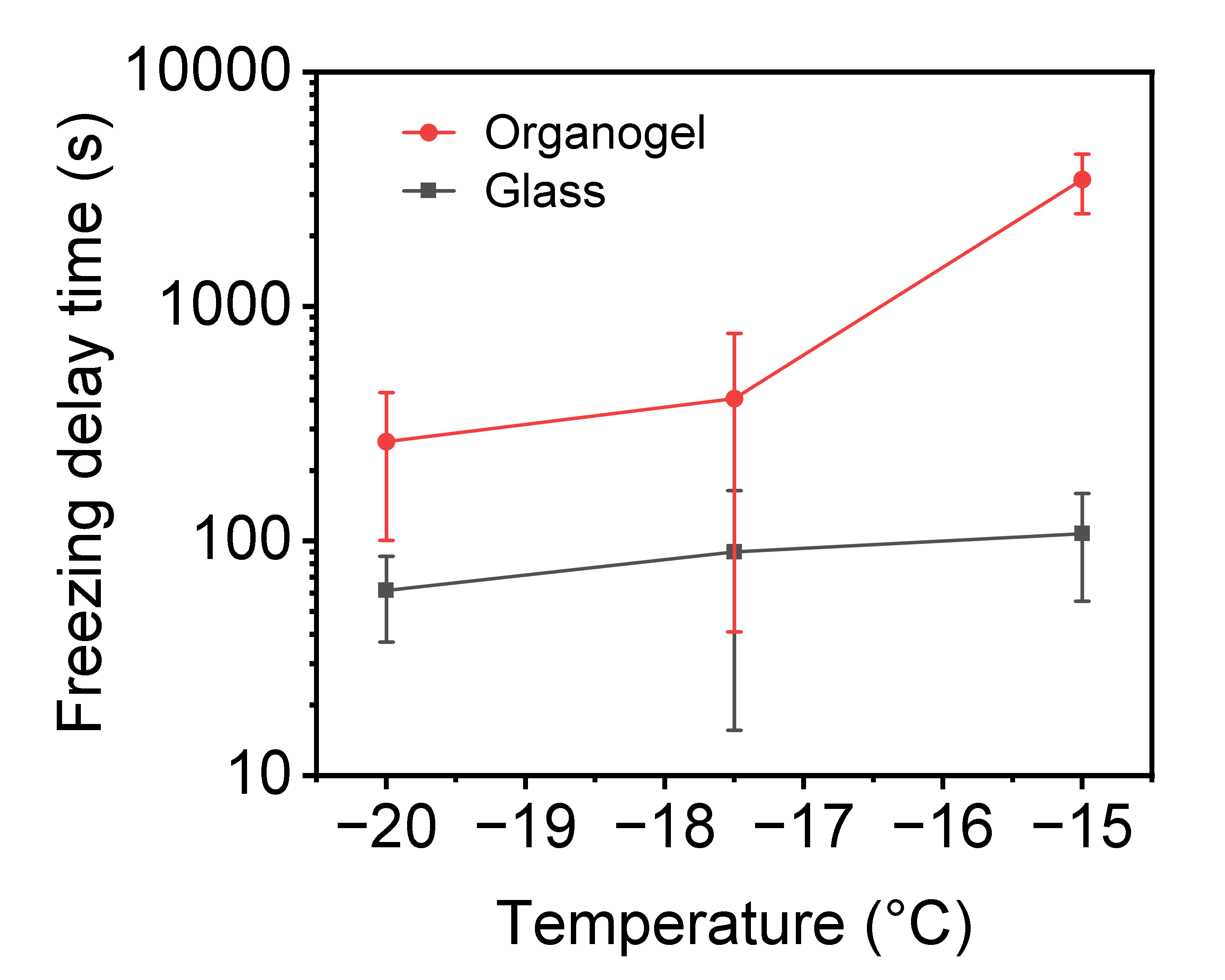
**

Figure S5. Freezing delay times of water droplet on the organogel and glass surface at surface temperatures of -15, -17.5 and -20 °C


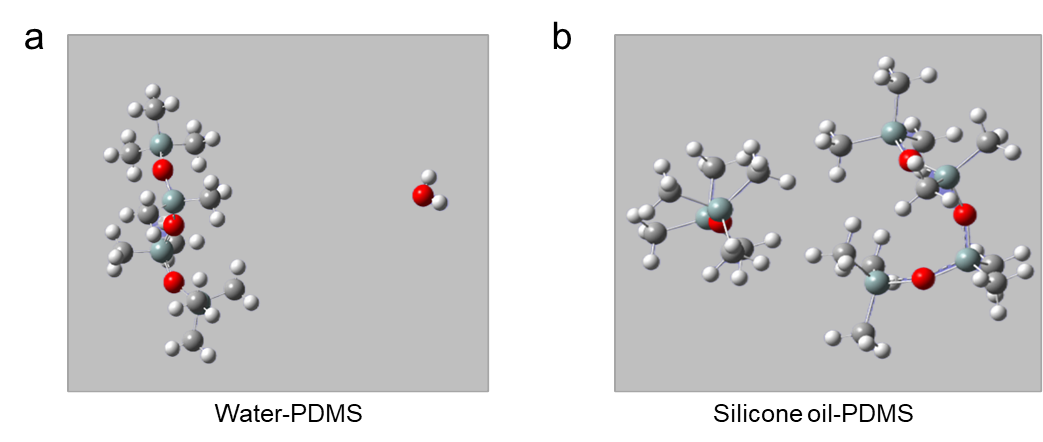


**Figure S6.** Diagrams of interactions between (a) water and PDMS, (b) silicone oil and PDMS


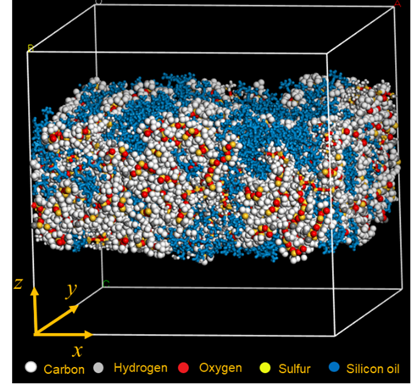


**Figure S7.** Molecular dynamics simulation box of the organogel.


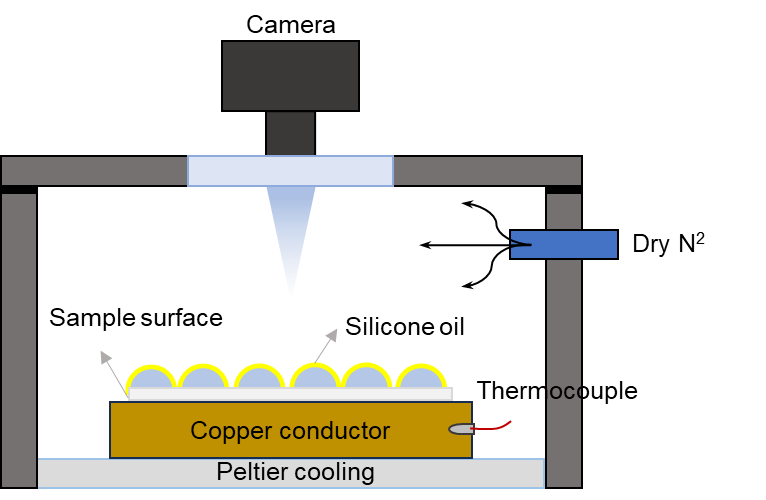


**Figure S8.** Experimental setup of freezing point measurement on surfaces


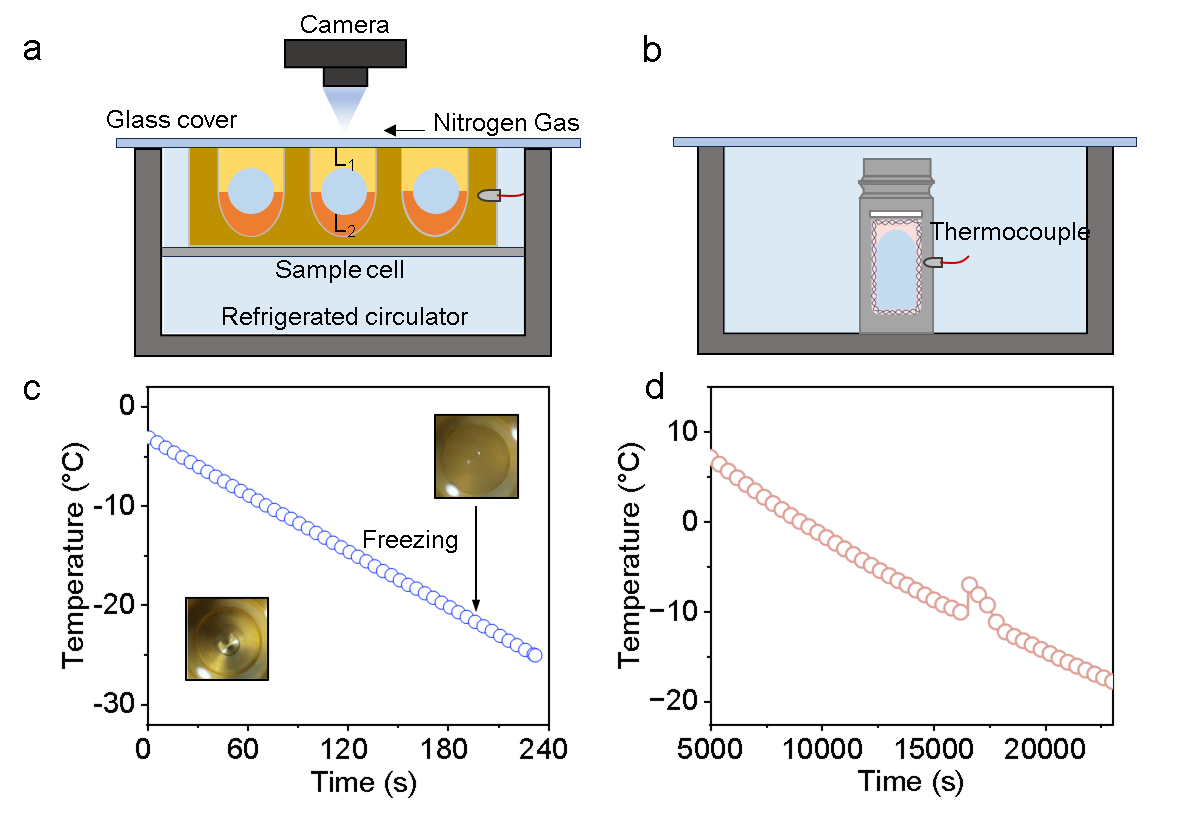


**Figure S9.** Supercooling degree measurement. (a) Experimental setup of measuring the supercooling degrees of small-volume droplets. L_1_ and L_2_ represent the layers of dimethyl silicone oil and phenylmethyl silicone oil layer, respectively. (b) Experimental setup for measuring the supercooling degree of large-volume solution. (c) Droplet temperature as a function of running time. The nucleation event is indicated by a change in droplet optical transparency, with inset images captured before and immediately after nucleation. (d) Solution temperature as a function of running time, where the nucleation process is identified by a significant temperature jump.


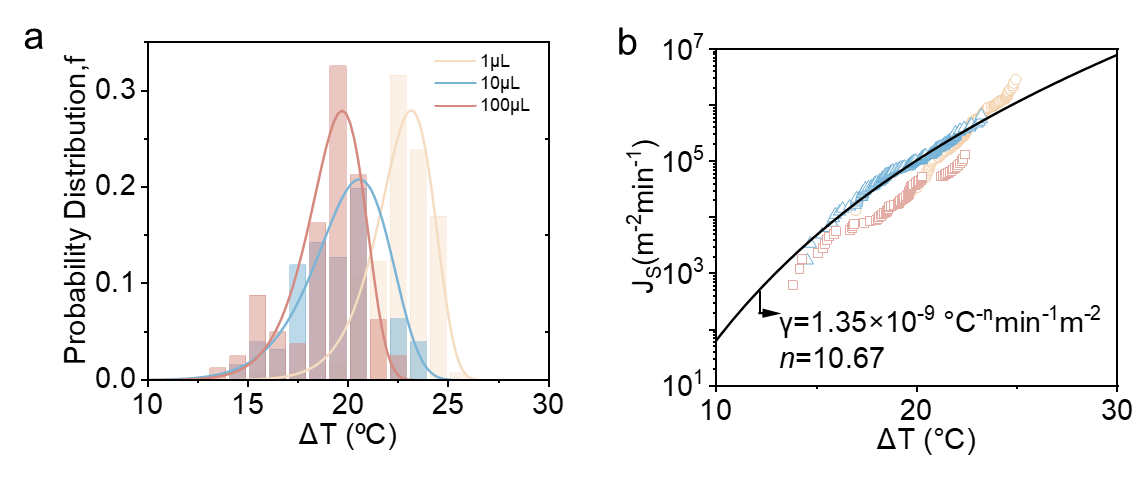


**Figure S10.** Probability distribution and nucleation parameter fitting. (a) Probability distribution of water droplets with varying volumes. (b) Nucleation rate of water droplets with different volumes. The parameters *γ* and *n* represent the empirical constants fitted by experimental data from Fig. S8a.


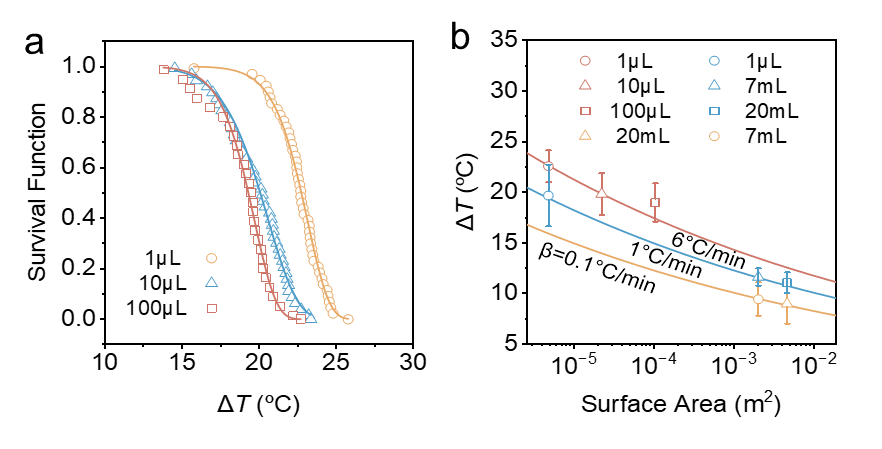


**Figure S11.** Supercooling prediction of large-volume of water. a) Survival function of water droplets with varying volumes. b) Comparison of supercooling degrees between statistical predictions (dash line) and experimental data (scatter points) for different volumes of water, highlighting the accuracy of the predictive model.


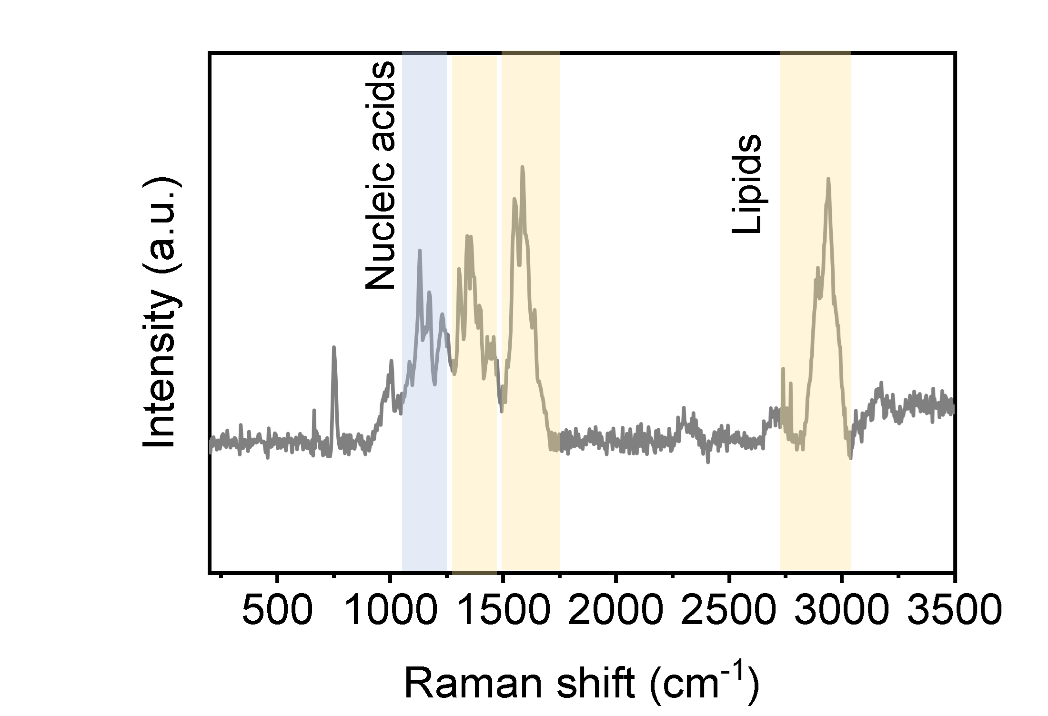


**Figure S12.** Raman spectrum of organ surface. The peaks at 1360 cm⁻¹, 1550 cm⁻¹, and 2850 cm⁻¹ represent characteristic Raman peaks of lipids.


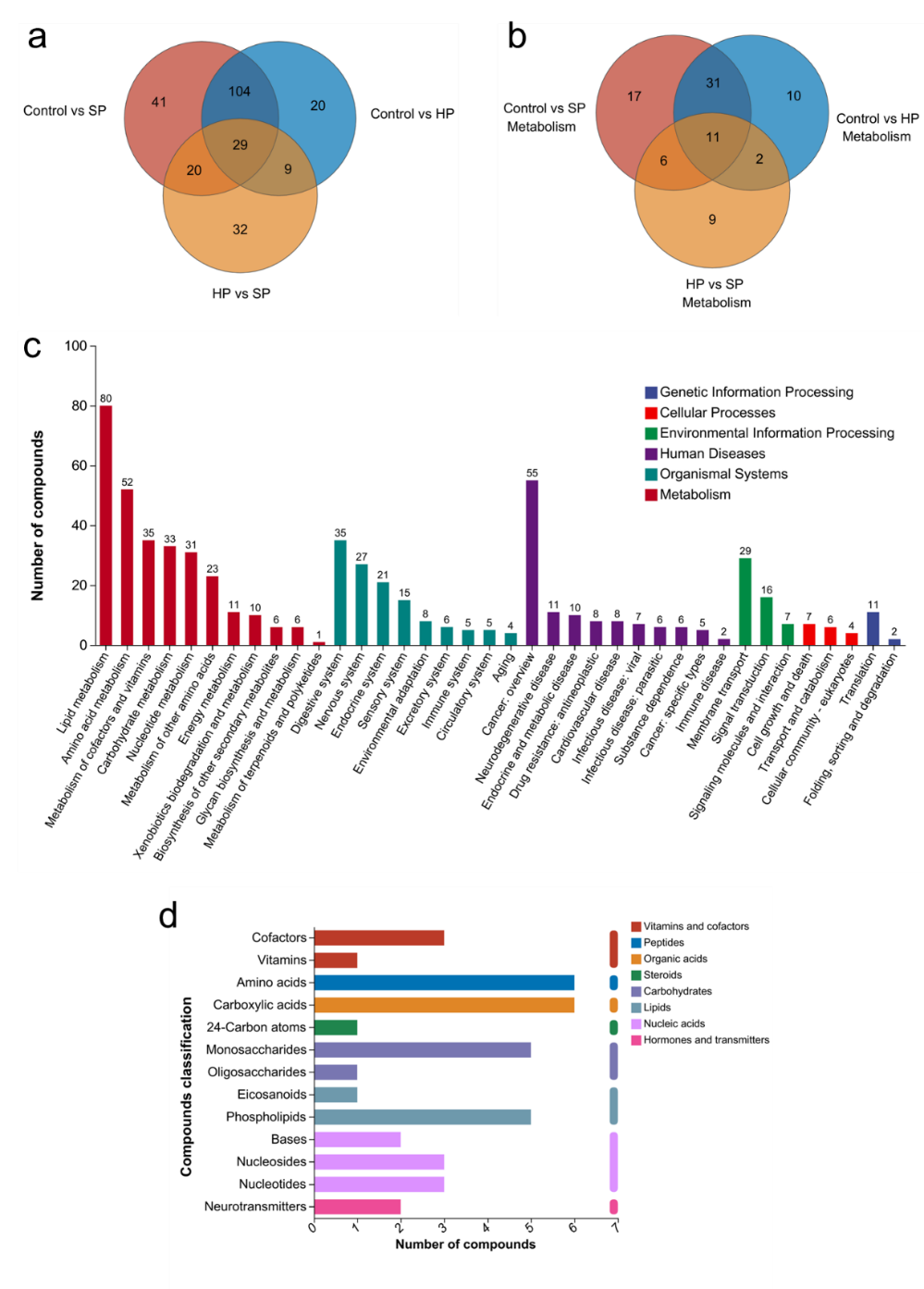


**Figure S13.** Untargeted Metabolomics of hearts preserved for 24 h. (a) Venn diagram of the total metabolites. (b) Venn diagram of the differential metabolites that were enriched to metabolic functions. (c) KEGG pathway in which the total metabolites were mainly enriched. (d) Classification of the differential metabolites, which predominantly included amino acids, monosaccharides, phospholipids, carboxylic acids, and so on.


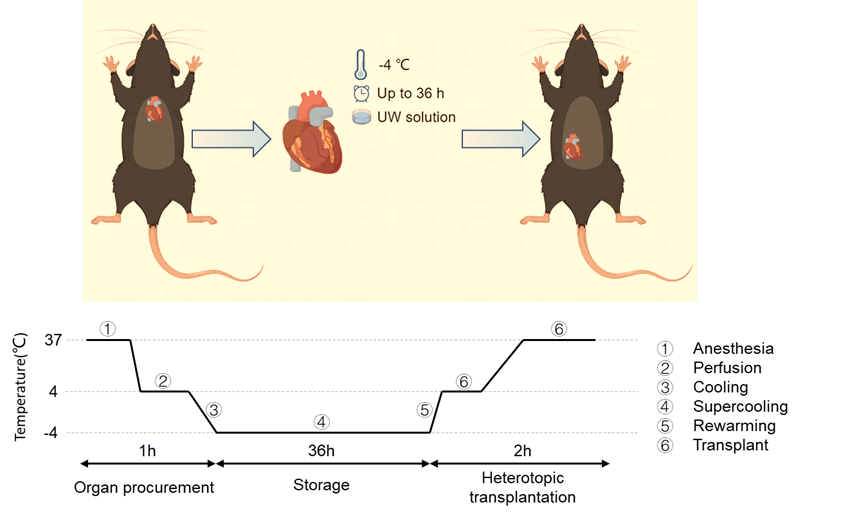


**Figure S14.** Experimental procedure of supercooling preservation and transplantation. (1) Mice were anesthetized with pentobarbital sodium (60 mg/kg) administered via intraperitoneal injection to ensure a controlled surgical environment. (2) Hearts were perfused with University of Wisconsin (UW) solution through the aorta to facilitate preservation, followed by surgical procurement of the hearts. (3) The harvested hearts were cooled to -4 °C. (4) The hearts were maintained at -4 °C for a total of 36 hours. The total volume of the storage system was about 5ml. (5) The hearts underwent rapid rewarming to 4 °C. (6) End-to-side anastomosis was performed, connecting the donor aorta to the recipient's abdominal aorta, and the donor pulmonary artery to the recipient's subrenal inferior vena cava to establish heterotopic heart transplantation.


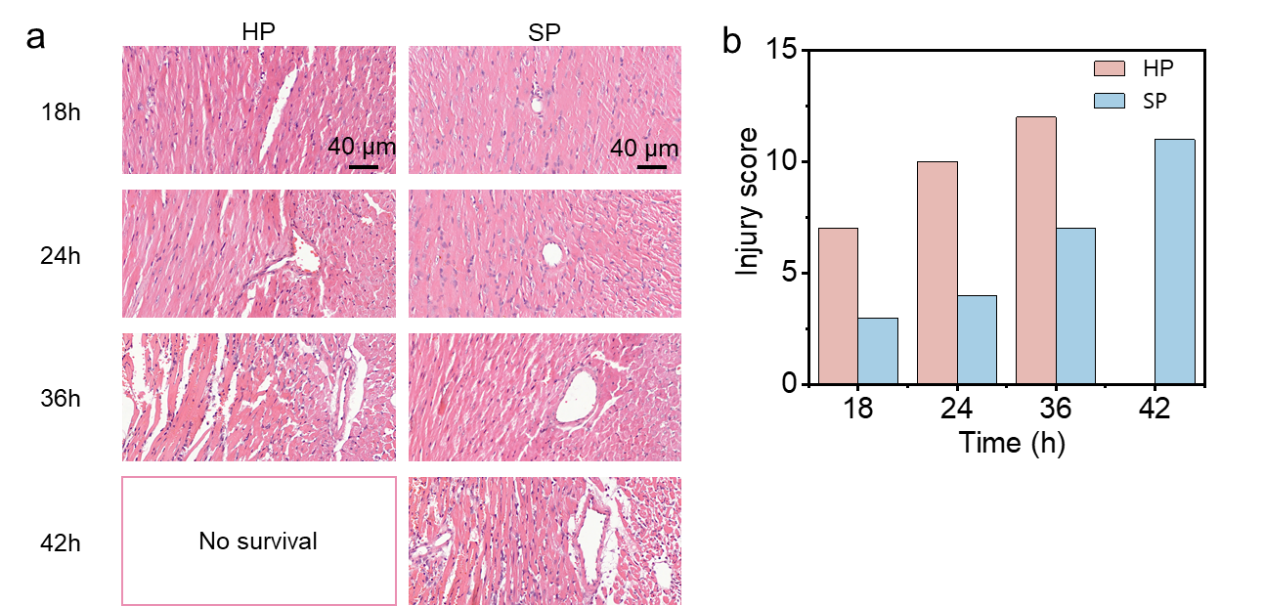


**Figure S15.** Histological injury of hearts 24 h after transplantation. (a) Representative Hematoxylin and Eosin (HE) staining images of hearts after 24 hours of transplantation. The images showcase hearts that were preserved by HP or SP for varying durations: 18 hours, 24 hours, 36 hours, and 42 hours. (b) Injury scores for each group, which were calculated based on the histological assessment. The data indicates the level of cardiac injury correlated with the preservation temperature and duration.


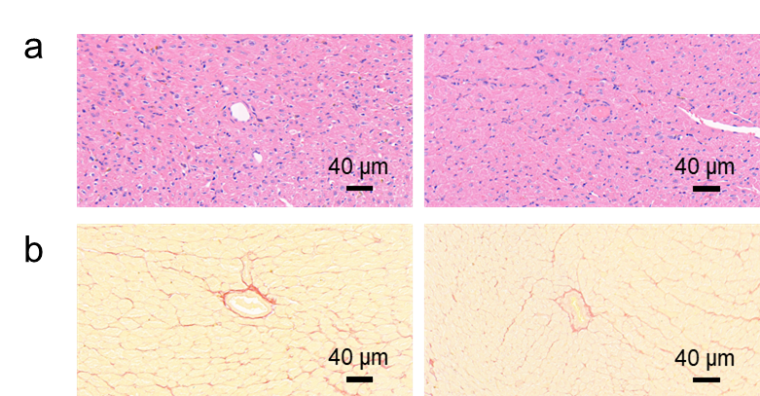


**Figure S16.** Histological injury and fibrosis level of hearts after transplantation for 3 months. (a) Representative Hematoxylin and Eosin (HE) staining images of hearts after 3 months of transplantation. The images compare hearts by HP for 18 hours versus those by SP for 36 hours. (b) Representative Sirius Red staining images of hearts after 3 months of transplantation. This staining method specifically highlights collagen deposition and fibrosis levels in the heart tissue. Sample size for each group is *n* = 6

| Year | Reference | Method | Preservation Conditions | Performance | Disadvantages |
| --- | --- | --- | --- | --- | --- |
| 2018 | Wan et.al^[27]^ | Isochoric | Rat heart:  -8°C/78 MPa/1 h; | No transplantation data; cardiac tissue damage in vitro was superior to the control group. | High hydrostatic pressure causes damage |
| 2021 | Powell-Palm et.al^[28]^ |  | Cardiac microtissues:  -4°C/72 h | No transplantation data; higher heartbeat rate and spontaneous beating rate than control. | Limited to tissue preservation |
| 2014 | Berendsen et.al^[8a]^ | Machine perfusion | Rat liver:  -6°C/72 h; | All recipients survived after 72-hour preservation, but survival dropped to 60% at 96 hours. | Relying on a large amount of cryoprotectants;  Complex system setup |
| 2019 | de Vries et.al^[9]^ |  | Human liver:  -4°C/20 h; | No transplantation data; Cold-preserved liver met clinical transplant standards in bile pH, glucose, and apoptosis. |  |
| 2020 | Que et.al^[11]^ |  | Mouse heart:  -6°C/144 h | Transplant group showed better outcomes in: myocardial IRI, oxidative stress, and apoptosis vs control. |  |
| 2001 | Takahashi et.al^[29]^ | High-pressure preservation | Rat liver:  5 MPa/-2°C/5 h | Following 5-hour preservation, liver transplantation demonstrated satisfactory postoperative hepatic function; however, recipient survival rates decreased when portal pressure exceeded 10 MPa. | High pressure compromise organ quality |
| 2025 | This work | Organogel | Mouse heart:  -4°C/36 h | The preservation time of the mice was doubled to 36 hours, and the degree of myocardial damage and fibrosis after transplantation was better than that of the control group. | - |

**Table S1.** Comparison of existing supercooling strategies
